# Effect of Fatty Acid Unsaturation on Phytosteryl Ester Degradation

**DOI:** 10.1007/s11746-017-2979-x

**Published:** 2017-04-01

**Authors:** Marianna Raczyk, Dominik Kmiecik, Roman Przybylski, Magdalena Rudzińska

**Affiliations:** 1 Faculty of Food Science and Nutrition, Poznań University of Life Sciences, Wojska Polskiego 31, 60-624 Poznań, Poland; 2 Department of Chemistry and Biochemistry, University of Lethbridge, Lethbridge, Canada

**Keywords:** Stigmasterol, Fatty acid, Ester, Heating, Degradation

## Abstract

This study examined the thermo-oxidative degradation of stigmasterol fatty acids esters. Stigmasterol stearate, oleate, linoleate and linolenate were synthesized by chemical esterification and their purity evaluated by ^1^H-NMR and GC–MS. The degradation of stigmasterol esters was examined after heating them at 60 and 180 °C for 1, 2, 4, 8 and 12 h. It was established that stigmasterol esters were prone to thermo-oxidative degradation, with time and temperature affecting the degree of degradation. The unsaturation of fatty acids affected the rate of stigmasteryl ester degradation. The kinetics of StS and StO degradation were similar and the additional double bonds in StL and StLn resulted in their faster decomposition. The esters degraded faster at 180 than at 60 °C. The sterol and fatty acid molecules degraded at different rates, such that the fatty acid moiety deteriorated faster than the sterol at both temperatures, independent of the time of heating and the level of unsaturation.

## Introduction

Phytosterols (PS) are derived exclusively from plant sources and perform similar cellular functions in plants to cholesterol in humans and animals. The richest sources of plant sterols are vegetable oils, oilseeds, nuts, fruits, and vegetables. Over 200 sterols have been identified in plant matter, of which the most common are sitosterol, campesterol and stigmasterol [1].

Phytosterols, in their free and esterified forms, are added as functional compounds to food products, due to their blood cholesterol-lowering efficacy [2]. Clinical studies have demonstrated that phytosterols and their esters lower serum cholesterol levels at similar rates [1]. The consumption of 3.6 g of phytosteryl ester (PE) daily was shown to result in a 9.7% decrease in LDL cholesterol and a 7.3% decrease in TAG, but did not alter HDL cholesterol [3]. The benefits of cholesterol lowering were observed at a similar rate in individuals with hypercholesterolemia, mildly and moderately elevated blood cholesterol, and coronary heart disease [4]. Due to their solubility, plant steryl esters are well tolerated and can usually be added in higher amounts to high-fat food products such as margarines and spreads. Steryl esters incorporated into a low-fat low-cholesterol diet showed better efficacy in lowering blood cholesterol lipids than the diet alone [5].

The enrichment of food products with phytosterols is difficult because of their poor solubility in fat and their insolubility in water. The esterification of free sterols improves their fat solubility [6]. The plant sterols currently incorporated into foods are esterified to unsaturated fatty acids to increase their lipid solubility and to facilitate their incorporation into foods with limited amounts of fat [7]. The levels of added phytosterols or phytostanols are usually similar to the recommended daily intake of 2–3 g sterol compounds per serving portion [8]. Currently, more than 40 patents deal with phytosterol-enriched food products and more than 15 commercial products are currently marketed worldwide [9]. Products enriched with plant sterols are offered as functional foods in the form of margarines, spreads, sausages, dressings, yogurts, milk and bread.

Some of these products are used for thermal food processing, such as frying, baking, and cooking which can stimulate thermo-oxidative degradation. This can lead in consequence to the formation of detrimental phytosterol and fatty acid oxidation products, as well as of oligomers such as dimers and trimers and the creation of off-flavor volatile compounds [10].

The main source of endogenous fatty acid sterol esters is a soybean oil deodorization distillate [11], which is usually purified and concentrated by molecular distillation using temperatures of 180 and 250 °C [12]. These elevated temperatures may stimulate or initiate thermo-oxidative degradation potentially creating free radicals. The other most common source of sterol esters is the chemical esterification between free sterols and fatty acids. Both substrates are usually obtained from deodorization distillates formed during the processing of commodity oils such as rapeseed/canola, soybean and sunflower oil. Tall oil, a by-product of the wood pulping industry, is a rich source of plant sterols [13].

During the thermo-oxidative degradation of vegetable oils, such as frying, polar compounds are formed as the result of oxidation, polymerization and hydrolysis. The formation of polar compounds is strongly related to the formation of primary and secondary oxidation products including monomeric oxidized products, as well as dimers and oligomers of TAGs and other endogenous oil/fat components, including sterols and steryl esters [14]. The total amount of polar compounds is a very useful indicator of frying oil quality, as is noted in many international regulations [15]. The concentration of total polar compounds in frying oil is regulated in many European countries for public health reasons [16].

Oxidized phytosterols (POX) are formed during the storage and processing of food, and have been observed in human serum, indicating absorption from the gut [17]. These compounds are common in dried canola seeds, refined and cold-pressed oils, French fries, spread enriched with phytosterols, potato chips, infant formulas, coffees, and heated oils, which substantiates the easy with which phytosterols undergo oxidative degradation [18].

POX, similarly to cholesterol oxidation products, have atherogenic and inflammatory properties, some published data suggesting that dietary POX can increase the risk of formation of atherosclerotic lesions and plugs [18]. A better understanding of how POX and other detrimental components are formed during food processing, preparation and storage is required [19].

The main goal of this study was to establish the pattern of thermo-oxidative degradation of fatty acid esters of stigmasterol during accelerated storage (60 °C) and during thermal treatment such as frying (180 °C).

## Materials and Methods

### Materials

Stigmasterol (≥95%), stearic acid (≥98.5%), oleic acid (≥99%), linoleic acid (≥99%), linolenic acid (≥99%), all solvents, sodium hydroxide, anhydrous pyridine, the catalysts-dicyclohexylcarbodiimide (DCC) and 4-dimethylaminopyridine (DMAP), high-purity silica gel 70–230 mesh, standards of cholesteryl stearate (96%), oleate (98%), linoleate (98%), linolenate (97%), 5α-cholestane and heptadecanoic acid methyl ester were purchased from Sigma-Aldrich (St. Louis, MO, USA). The silylation mixture Sylon BTZ was supplied by Fluka Analytical (Buchs, Switzerland). The internal standard 19-hydroxycholesterol was purchased from Steraloids (Newport, RI, USA). The silylation mixture of BSTFA [*N*,*O*-Bis(trimethylsilyl) trifluoroacetamide] with 1% TMCS (trimethylchlorosilane) was obtained from Fluka Chemie, while the SEP-PAK amino cartridges were sourced from Waters (Milford, USA).

### Esterification

Chemical esterification [20] was used to obtain the esters of stearic, oleic, linoleic and linolenic acids and stigmasterol. Briefly, 500 mg of stigmasterol was dissolved in 30 ml of dichloromethane and placed in a three-necked flask. The air was replaced by argon and the catalysts (500 mg DCC and 15 mg DMAP) and 600 mg of fatty acid were added. Esterification was run at room temperature for 24 h in the dark. The reaction mixture was then placed in a separatory funnel, 10 ml of distilled water was added, and the entire mixture was shaken. The lower layer was collected in the flask. The cleaning procedure was repeated three times. The solvent from the collected fractions was evaporated under vacuum at 30 °C and the residue was dissolved in 20 ml of hexane. The esterified mixture was further cleaned using a silica gel column (45 × 2.5 cm), eluting the clean fraction with 450 ml of hexane:ethyl acetate (9:1, v/v). The purity of the fraction was verified by TLC, comparing it with the cholesteryl oleate standard. The quality of esterified esters was determined using ^1^H-NMR and GC–MS. Due to the small amount of material, we did not calculate the precise yield of the esterification, but approximately 500 mg steryl ester was obtained.

### GC-EI-MS Identification of Stigmasteryl Esters

The identity was confirmed using an Agilent Technologies 7890A GC coupled to a 5975C VL MSD Triple-Axis Detector after separation on a DB-5 capillary column (30 m × 0.2 mm, 0.32 mm; J&W Scientific, Folsom, CA, USA). Helium was used as a carrier gas at a flow rate of 0.6 ml min^−1^. Mass spectra were recorded using electron-impact ionization mode at 15 and 70 eV and scanning mass was in the range of 100–650 Da. The ion source was held at 200 °C and the injector at 300 °C. A combination of the NIST Mass Spectra Library and a cholesteryl ester mass spectrum was used for identification.

### Sample Heating

Stigmasteryl esters (500 mg) were placed into a 200-ml glass ampoule before sealing, 100 ml of pure oxygen was slowly injected to provide a surplus of oxygen, preventing oxygen starvation. Samples were heated at 60 or 180 °C and kept at those temperatures for 1, 2, 4, 8 and 12 h. A separate ampoule was supplied for each time point. The heating experiment was duplicated. After the heating ampoules were cooled to room temperature, they were placed in liquid nitrogen and stored for analysis, though not longer than 24 h. The samples were warmed to room temperature, dissolved in 10 ml of chloroform:methanol (1:1, v/v) and immediately analyzed. The kind of solvent was chosen experimentally, because samples heated at 180 °C for 8 and 12 h were insoluble in such organic solvents as tetrahydrofuran, methyl-*tert*-butyl-ether, hexane and chloroform. The data presented here are averages from the two experiments. An unheated sample of esters was used as the control.

### GC-FID Analysis of Stigmasteryl Esters

The quantity analysis of the esters was performed according to Barnsteiner et al. [21]. Briefly, 1 mg of ester was dissolved in chloroform:methanol (2:1, v/v) and 1 μl of prepared sample was injected into an Agilent Technologies Instrument 6890 SII equipped with a flame ionization detector. For separation, an Rtx-200MS capillary column (30 m × 0.25 mm × 0.1 μm; Restek, Bad Homburg, Germany) was used. The temperatures of inlet and detector were 280 and 360 °C, respectively. Hydrogen was used as a carrier gas with a constant flow of 1.5 ml min^−1^. The sample was injected in splitless mode. The oven temperature was programmed from 100 to 310 °C at a rate of 15 °C min^−1^, to 315 °C at a rate of 1.5 °C min^−1^ and to 340 °C at a rate of 15 °C min^−1^. At 310 and 340 °C, the temperature was constant for 2 min. The quantification was performed using cholesteryl oleate as an internal standard, with a relative response factor of 1.00. The samples from an autonomous series were analyzed in triplicate.

### Determination of Stigmasterol Moiety

The content of the stigmasterol moiety was determined using AOCS Official Method Ch 6-91 [22]. An amount of 1 mg of ester was saponified with 1 M KOH in methanol for 18 h at room temperature, water was then added and the unsaponifiables extracted three times with hexane/methyl *tert* butyl ether (1:1, v/v). The solvent was evaporated under a stream of nitrogen and the the residues dissolved in 0.1 ml pyridine before silylation with 0.4 ml of Sylon BTZ (Supelco, Bellefonte, PA, USA). The derivatives of the sterol were separated on a gas chromatograph HP 6890 equipped with a DB-35MS capillary column (25 m × 0.20 mm; 0.33 μm; J&W Scientific). A sample was injected in splitless mode. The column temperature was held at 100 °C for 5 min, then programmed to 250 °C at a rate of 25 °C min^−1^, held for 1 min, and further programmed to 290 °C at a rate of 3 °C min^−1^, with the final temperature held for 20 min. The detector temperature was set to 300 °C. Hydrogen was used as a carrier gas at a flow rate of 1.5 ml min^−1^. 5α-Cholestane was used as the internal standard for sterol quantification with a general relative response factor 1.00, which was confirmed using commercially available stigmasterol standard. Stigmasterol was identified and quantified by a comparison with the retention data of the standard.

### Determination of Fatty Acid Moieties

Stigmasteryl esters before and after heating (0.1 g) were placed in glass vials and hydrolyzed with 0.5 M KOH in methanol. Fatty acids were methylated with 14% boron trifluoride in methanol as the catalyst, in line with AOCS Official Method Ce 1 k-07 [23]. Fatty acid methyl esters were analyzed using a 5890 Series II gas chromatograph from Hewlett-Packard (Palo Alto, CA, USA) equipped with a Supelcowax 10 capillary column (30 m × 0.25 mm × 0.25 µm; Supelco). Samples were run in splitless mode, and the injector and detector were held at 240 °C. The column temperature was isothermal at 210 °C. Hydrogen was used as the carrier gas at a flow rate of 1.0 ml/min. The quantification of fatty acids was performed using methyl heptadecanoate as the internal standard. The comparison of retention data of fatty acid methyl ester standards was used for identification.

### Total Polar Compounds (TPC)

The total polar compounds were determined according to DGF Standard Method C-III 3e [24]. Two fractions were collected: (1) the nonpolar fraction, eluted with THF, contained 250 ppm BHT as antioxidant; and (2) the polar fraction was eluted using isopropyl ether.

### Stigmasterol Oxidation Products (StOX)

Stigmasterol oxides were determined after transesterification, followed by SPE fractionation and GC-FID separation, in line with to the procedure described by Rudzińska et al. [25]. Briefly, 0.1 g of stigmasteryl esters, 20 µg of internal standard (19-hydroxycholesterol) and 2 ml of sodium methoxide (10% in methanol) were added and vortexed. The oxysterol fraction was extracted with chloroform and evaporated to dryness under a stream of nitrogen. The residue was dissolved in 250 µl of chloroform and fractionated using a SEP-PAK NH2 cartridge. The final fraction of StOX was removed by acetone, the solvent was evaporated and the residue was derivatized by BSTFA+ 1% TMCS. StOX derivatives were analyzed on a Hewlett-Packard 6890 gas chromatograph equipped with a DB-5MS column (30 m × 0.25 mm × 0.25 mm; J&W Scientific). Samples were injected in splitless mode and the column temperature was programmed as follows: the initial temperature of 160 °C was held for 1 min, then programmed at 40 °C/min to reach 270 °C, where it was held for 1 min; it was further programmed at 4 °C/min to 280 °C, with this final temperature being held for 25 min. Hydrogen carrier gas at a flow rate of 1 ml/min was used. The detector and injector temperatures were 300 °C.

The oxidized derivatives were identified on a 7890A GC system (Agilent Technologies) coupled to a 5975C VL Triple-Axis mass detector (Agilent Technologies) using the column and conditions described above. All mass spectra were recorded using electron impact ionization mode at 70 eV and the masses were scanned from 100 to 650 Da. The ion source was held at 200 °C and the injector at 300 °C. To identify the compounds, a combination of NIST Mass Spectra Library, our laboratory library of collected oxyphytosterol data, and the retention data of the standards were utilized. The peaks which were not identified are calculated together and are presented as a sum named “others”.

### Statistics

Each result shown in the tables represents the mean of both experiments and of the three replicate analyses for each sample. Statistical analysis was done by one-way analysis of variance (one-way ANOVA) in Statistica 10.0 software. Differences at *P* < 0.05 were considered significant.

## Results and Discussion

### Identification of Stigmasteryl Esters

**Fig. 1:**
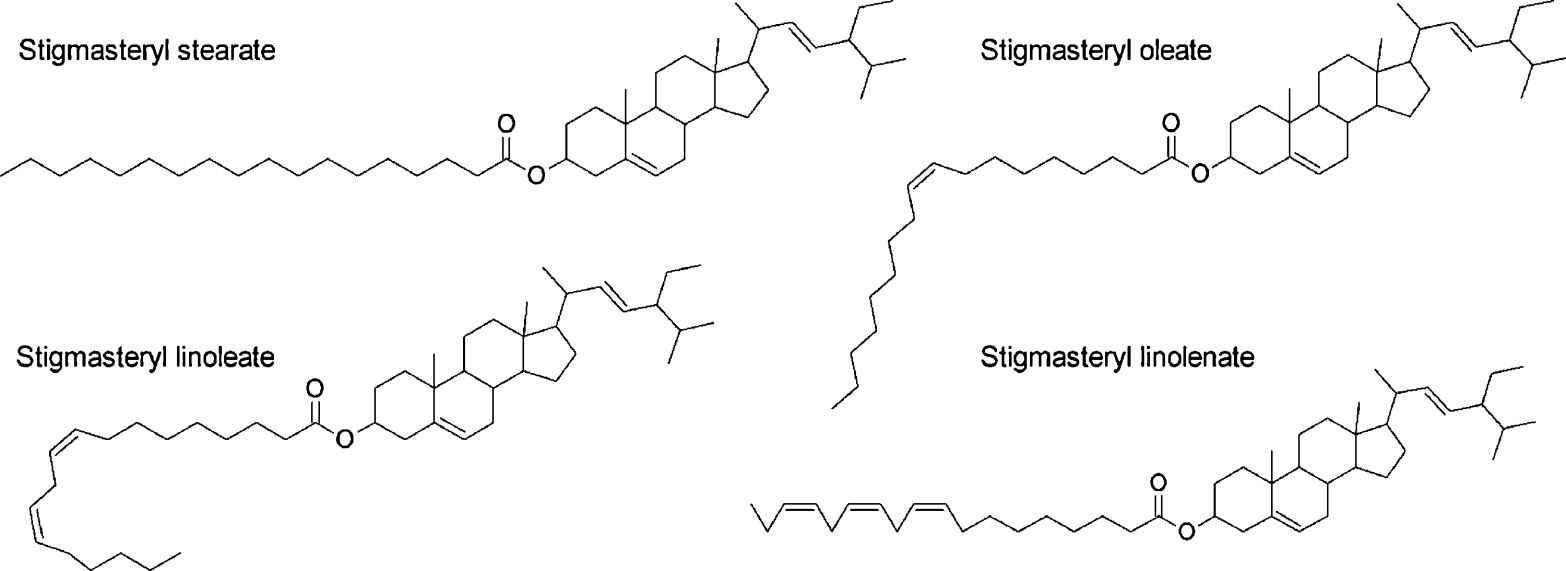
Chemical structures of stigmasteryl esters

**Fig. 2:**
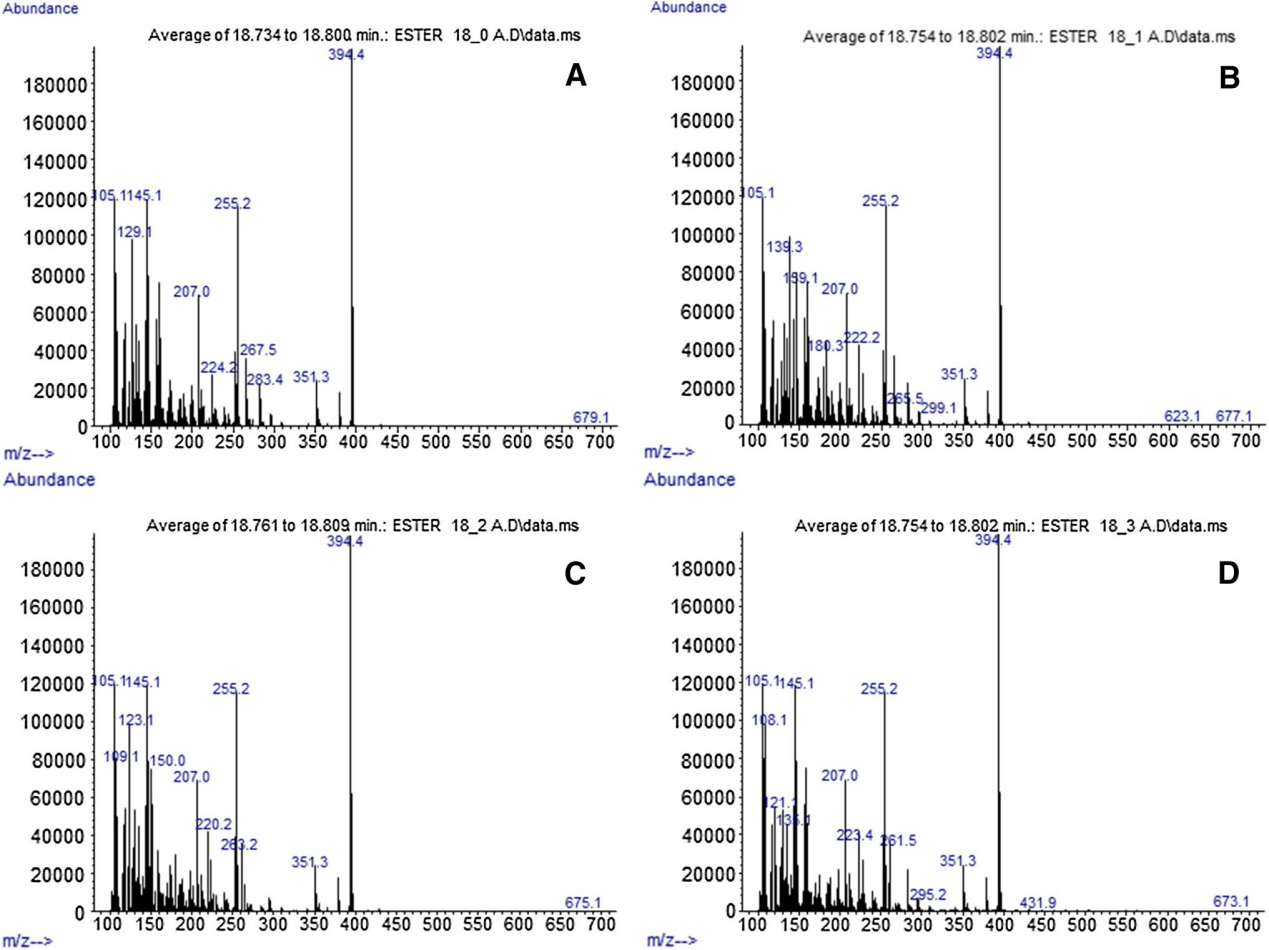
Mass spectra of: **a** stigmasteryl stearate, **b** stigmasteryl oleate, **c** stigmasteryl linoleate, **d** stigmasteryl linolenate

Phytosteryl esters are not commercially available, though the synthesis of stearic, oleic, linoleic and linolenic esters of stigmasterol has been executed. The chemical structures of the synthetized stigmasteryl esters in Fig. 1 are presented, followed by their mass spectra in Fig. 2. The molecular ions of the esters were not observed, but the stigmasterol moiety provided the characteristic fragments at *m*/*z* = 394, 351 and 255 (Table 1). The fragment with *m*/*z* = 394 originated from the loss of fatty acid molecule with two additional oxygen atoms. The fragment with *m*/*z* = 351 was formed after partial fragmentation of the sterol side chain by the removal of the C3H7 unit. Identification of the fatty acid moiety is based on the ratio between the fragment with *m*/*z* = 74 to *m*/*z* = 87 and *m*/*z*= 81 [26]. The fragmentation of stearic acid formed ions with *m*/*z* = 267 and 129 characteristic of this acid methyl ester, which is absent from the mass spectra of the unsaturated C18 fatty acids used in this study. The ion with *m*/*z* = 227 represents the rearrangement of a three-carbon atom unit formed from the second to fourth carbon atom on the fatty acid chain (Christie 2013, http://lipidlibrary.aocs.org).

**Table 1:** Typical fragments formed from stigmasteryl esters

Steryl esters	Mass of ions typical for	
	Sterol moiety fragments	Fatty acid moiety fragments
Stigmasteryl stearate C_47_H_82_O_2_ MW = 679	394 (M-C18H35O2)^+^, 351 (M-C18H35O2-C3H7)^+^, 255 (M-C18H35O2-C10H19)^+^	283 (M-C29H48)^+^, 267 (M-C29H48-O)^+^ 224 (M-C29H48-C3H6O)^+^ 129 (M-C29H48-C8H17O)^+^
Stigmasteryl oleate C_47_H_80_O_2_ MW = 677	394 (M-C18H33O2)^+^, 351 (M-C18H33O2-C3H7)^+^, 255 (M-C18H33O2-C10H19)^+^	281 (M-C29H48)^+^ 265 (M-C29H48-O)^+^ 222 (M-C3H6O)^+^ 180 (M-C6H13O)^+^
Stigmasteryl linoleate C_47_H_78_O_2_ MW = 675	394 (M-C18H31O2)^+^, 351 (M-C18H31O2-C3H7)^+^, 255 (M-C18H31O2-C10H19)^+^	279 (M-C29H48)^+^ 263 (M-C29H48-O)^+^ 220 (M-C29H48-C3H6O)^+^ 150 (M-C29H48-C7H12O2)^+^ 123 (M-C29H48-C9H15O2)^+^
Stigmasteryl linolenate C_47_H_76_O_2_ MW = 673	394 (M-C18H29O2)^+^, 351 (M-C18H29O2-C3H7)^+^, 255 (M-C18H29O2-C10H19)^+^	277 (M)^+^ 261 (M-C29H48-O)^+^ 135 (M-C29H48-C8H14O2)^+^ 121 (M-C29H48-C9H16O2)^+^ 108 (M-C29H48-C10H16O2)^+^

The mass spectrum of stigmasteryl oleate showed ions with *m*/*z* values of 180, 222 and 265. The first fragment was formed by cleavage and hydrogen rearrangement on a fragment formed from the fifth and sixth carbon atom of the acid molecule (Christie 2013, lipidlibrary.aocs.org). The ion with *m*/*z* = 222 was formed by the loss of the McLafferty fragment (*m*/*z* = 74) from a fatty acid molecule. The characteristic sterol moiety ion with *m*/*z* = 255 was also detected (Table 1).

The mass spectrum of steryl linoleate shows ions typical of a steryl moiety fragment with m/z = 263, including the fragment with *m*/*z* = 220 formed by the McLafferty rearrangement. The fragments with *m*/*z* = 150 and 123 are related to the loss of C7H12O2 and C9H15O2 units from fatty acid molecules, respectively. The ion with *m*/*z* = 150 is a typical fragment for polyunsaturated fatty acids with adouble bond at the n-6 configuration (Christie 2013, lipidlibrary.aocs.org).

The fragmentation of stigmasteryl linolenate was exhibited by the fragment with *m*/*z* = 108 characteristic of the removal of the n-3 terminal group, typical of a fatty acid with this configuration. The ions with *m*/*z* = 121 and 135 are associated with the cleavage of C9H16O2 and C8H14O2 units from acid molecule, respectively. The ion with *m*/*z* = 261 is typical of the loss of the sterol moiety from this ester molecule. The McLafferty fragment is always small in the mass spectra of trienoic acids whereas hydrocarbon fragments with the general formula [CnH(2n-5)]^+^ dominate (Table 1) (Christie 2013, lipidlibrary.aocs.org).

### Thermal degradation of Stigmasteryl Esters

The chosen temperatures respectively imitate the accelerated Schaal oven storage test and frying conditions. Determining their changes in complex food matrix is difficult and is rarely conclusive. Model studies are always well controlled and allowed for better observation of the processes occurring during thermo-oxidative degradation of stigmasteryl esters.

**Fig. 3:**
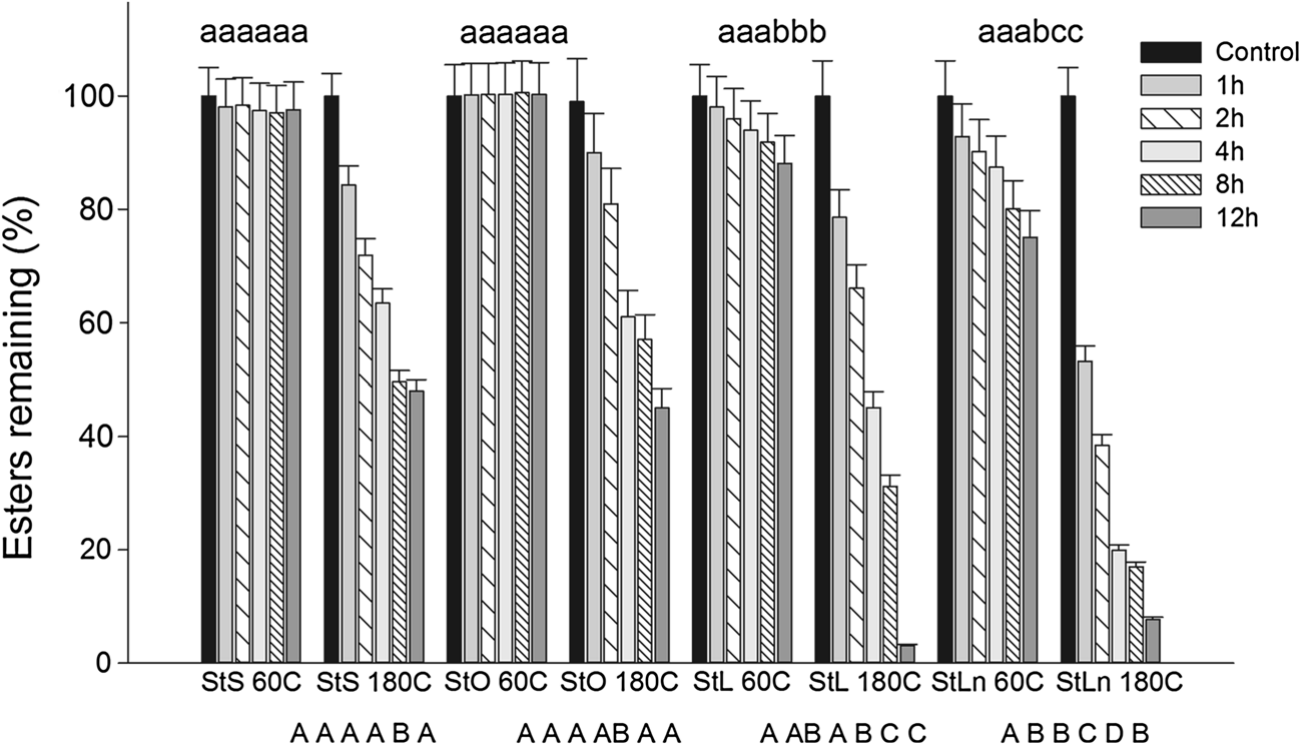
Degradation of stigmasteryl esters during heating at 60 °C and 180 °C for up to12 h; *StS* stigmasteryl stearate, *StO* stigmasteryl oleate, *StL* stigmasteryl linoleate, *StLn* stigmasteryl linolenate. Different *lowercase letters* above the bars denote statistically different degradation of different esters heated at 60 °C for the same time. Different *upercase letters* below the bars denote statistically different degradation of different esters heated at 180 °C for the same time

Ester degradation at 60 °C was much slower, even after 12 h of heating; here, 3% of the stearate ester and up to 25% of the linolenate ester disappeared (Fig. 3). As expected, heating at 180 °C caused much more rapid degradation after 12 h of heating, and 55 and 97% of stigmasteryl stearate and linolenate, respectively, had undergone degradation (Fig. 3). The rate of steryl ester decomposition was affected by the unsaturation of the fatty acids: negligible degradation was observed for stigmasteryl stearate (StS) and stigmasteryl oleate (StO) being heated at 60 °C for 12 h, whereas stigmasteryl linoleate (StL) and stigmasteryl linolenate (StLn) degraded faster, resulting in the loss of 12 and 25%, respectively (Fig. 3). After heating at 180 °C for 12 h, 52% of StS, 55% of StO, 97% of StL and 92% of StLn had disappeared (Fig. 3). At both temperatures, the stability of steryl esters was as follows: StS < StO < StL < StLn.

The thermo-oxidative degradation of steryl esters followed a first -rder kinetic curve where the *R* ^2^ was higher than 0.7 in all cases (Table 2). The kinetic constant values increased when the unsaturation of fatty acid and temperature increased (Table 2). For esters heated at 60 °C for 12 h, the kinetic constant value ranged from 0.00002 to 0.0005 min^−1^, while for the steryl and fatty acids moieties of the esters, the kinetic constant ranged from 0.0001 to 0.0012 min^−1^ and from 0.0001 to 0.0014 min^−1^, respectively. The kinetic constant for esters heated at 180 °C ranged from 0.0013 to 0.0053 min^−1^, whereas that for steryl and fatty acid moieties ranged from 0.0013 to 0.0043 min^−1^ and from 0.0008 to 0.0047 min^−1^, respectively. The kinetic constants further verified the relationship between the rate of degradation and the parameters of treatment, such that unsaturation and higher temperature caused faster degradation.

**Table 2:** Kinetic parameters for degradation of stigmasteryl esters, during heating at 60 and 180 °C for up to 12 h

Heated esters	Ester		Steryl moiety		Fatty acid moiety	
	*k* (min^−1^)	*R* ^2^	*k* (min^−1^)	*R* ^2^	*k* (min^−1^)	*R* ^2^
60 °C						
StS	0.0000a	0.7960	0.0001a	0.7380	0.0001a	0.7330
StO	0.0001a	0.7070	0.0003b	0.8273	0.0004b	0.7423
StL	0.0002b	0.8365	0.0005c	0.9333	0.0009c	0.9041
StLn	0.0005c	0.7075	0.0012d	0.9143	0.0014d	0.7733
180 °C						
StS	0.0009a	0.7105	0.0013a	0.8519	0.0008a	0.9474
StO	0.0013b	0.7969	0.0013a	0.7951	0.0016b	0.9026
StL	0.0036c	0.9929	0.0036b	0.9916	0.0026c	0.9833
StLn	0.0053d	0.9277	0.0043c	0.6929	0.0047d	0.9796

Different letters within the same column and temperature denote statistically different *k* values among samples

*StS* stigmasteryl stearate, *StO* stigmasteryl oleate, *StL* stigmasteryl linoleate, *StLn* stigmasteryl linolenate

First -rder kinetic model corresponding to

$$\ln \frac{{{\text{Ester}}_{t} }}{{{\text{Ester}}_{0} }} = - kt$$ or $$\ln \frac{{{\text{Steryl}}_{\text{t}} }}{{{\text{Steryl}}_{ 0} }} = - kt$$ or $$\ln \frac{{{\text{FA}}_{t} }}{{{\text{FA}}_{0} }} = - kt$$

When the influence of the degree of unsaturation of different lipid matrices (methyl esters of stearic, oleic, linoleic and linolenic acids) on a mixture of three plant sterols (campesterol, sitosterol and stigmasterol) at 180 °C for up to 3 h was performed, the kinetic constant value progressively decreased along with the degree of unsaturation [27]. Greater levels of unsaturation of the lipid matrix exhibited a protective effect against the degradation of plant sterols during heating, indicating that degradation of unsaturated fatty acids was easier than sterols [28]. This directly shows that the activation energy of degradation for unsaturated fatty acids is lower than that for sterols.

**Fig. 4:**
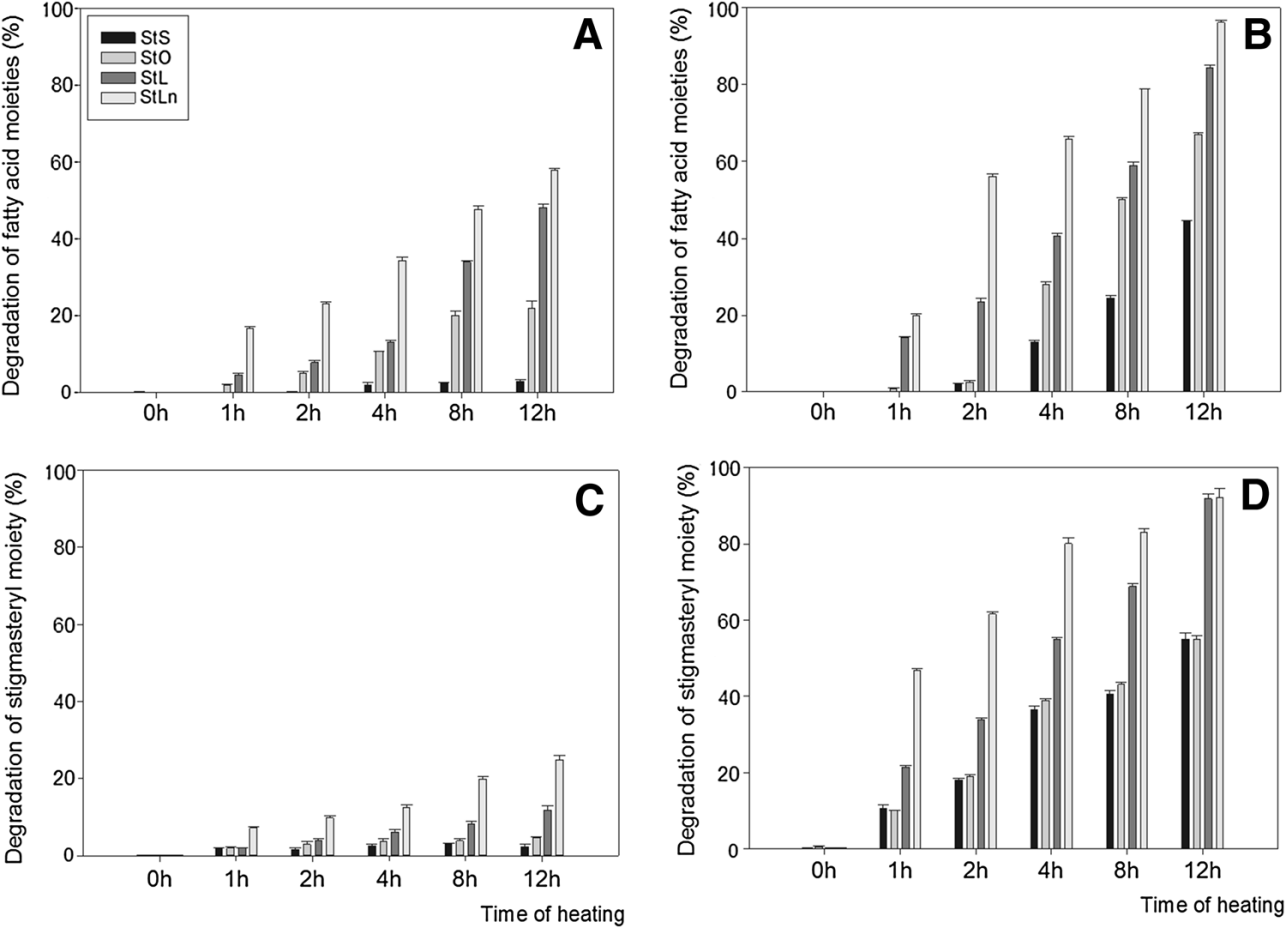
Degradation of fatty acid and stigmasterol moiety during heating of esters at 60 °C (**a**, **c**) and 180 °C (**b**, **d**)

The degradation process of steryl esters is the combined degradation reaction for the fatty acid and sterol moieties. When stigmasteryl esters are heated at 60 °C, the fatty acid part degrades at a higher rate than the sterol part. However, StS was an exception to this pattern when heated at this temperature for 4 h (Fig. 4). The opposite was observed during heating at 180 °C, where the degradation of the sterol moiety was faster than for fatty acids. In this case, StO was an exception when heated for 8 and 12 h, while StLn was the exception for 12 h of heating (Fig. 4). When esters were heated at 60 and 180 °C for 12 h, respectively, 3 and 44% of stearic acid, 22 and 87% of oleic acid, 48 and 84% of linoleic acid, and 58 and 96% of linolenic acid moieties disappeared. Unsaturation of the fatty acid moiety promoted sterol degradation, and, under heating at 60 °C, the degradation of esters increased with the unsaturation of the fatty acid moiety (Fig. 3). Even more drastic degradations were observed when esters were heated at higher temperatures, although the pattern of degradation was the same as for the lower temperature (Fig. 3). Higher unsaturation again caused faster degradation, indicating that free radicals formed fromthe oxidative decomposition of fatty acids stimulated the degradation of sterol. This clearly indicates that sterol degradation also follows the free radical pattern. Soupas et al. [29] established that fatty acid esters of phytosterols are more reactive than free phytosterols during prolonged heating at 100 °C; however, when the temperature increased to 180 °C, the opposite pattern of degradation was observed. Both the presence of an acyl moiety in the cholesteryl ester and its unsaturation affected the rate of cholesteryl molecule oxidation [30]. It has been shown that the presence of unsaturated fatty acids in a heated medium hindered phytosterol degradation [27]. Similar degradation levels to those found in our results were observed when stigmasterol was heated for 2 h at 180 °C in sunflower oil [31].

### Oxidation Products Formed During Degradation of Stigmasteryl Esters

The formation of polar compounds is a good indication of the thermo-oxidative degradation of lipids [32]. During thermo-oxidative degradation of stigmasteryl esters, the TPC increased constantly for the first 8 h of heating and then plateaued. The decreases in TPC under heating at 60 °C were 11.9% for StLn, 9.1% for StO, 7.6% for StL and 6.2% for StS. Under heating at 180 °C, the TPC was much higher and the rate of increase was very rapid after 1 h of heating (Table 3). After 8 h of heating at 180 °C, some decrease in the amount of polar components was observed. This decrease can be attributed to the degradation and interaction of polar compounds, since most of these are chemically very reactive [33]. Polar compounds in frying oils are composed of oligomers formed through the thermal polymerization of oxidation products such as aldehydes [34]. In addition, oxidized and nonoxidized free fatty acids formed through the cleavage of ester bonds can increase the polarity of food lipids [14]. The polar compounds can be formed from any lipid components present in the oil or fat, mainly through oxidative degradation, including from oxidation products formed from phytosterols.

**Table 3:** The changes of total polar compounds during heating of stigmasteryl esters (%)

Heated esters	Time of heating					
	Unheated	1 h	2 h	4 h	8 h	12 h
60 °C						
StS	1.0 ± 0.2a	1.9 ± 0.4a	5.9 ± 0.9a	6.4 ± 0.9a	6.2 ± 0.5a	5.6 ± 0.6a
StO	1.0 ± 0.2a	2.5 ± 0.1a	6.2 ± 0.4ab	8.4 ± 0.1ab	9.1 ± 0.1ab	7.2 ± 0.3a
StL	1.2 ± 0.1ab	5.7 ± 0.7c	5.6 ± 0.4a	6.4 ± 0.4a	7.6 ± 0.4a	7.1 ± 0.4a
StLn	1.7 ± 0.2b	4.3 ± 0.4b	8.4 ± 0.2b	7.1 ± 0.3b	11.9 ± 1.0b	11.1 ± 0.9b
180 °C						
StS	1.0 ± 0.2a	12.2 ± 0.9b	9.1 ± 0.7c	12.8 ± 0.3b	14.9 ± 0.8b	13.4 ± 0.6b
StO	0.9 ± 0.3a	15.3 ± 0.3a	16.9 ± 0.2a	17.2 ± 0.1c	28.6 ± 0.1a	18.2 ± 0.2a
StL	1.2 ± 0.2ab	19.6 ± 0.4c	20.5 ± 0.9b	23.5 ± 0.7a	24.9 ± 0.2c	17.2 ± 0.5a
StLn	1.8 ± 0.3b	15.1 ± 0.7a	19.1 ± 0.4ab	25.3 ± 0.3a	31.2 ± 0.6a	29.2 ± 1.0c

Different letters within the same column and temperature denote statistically different TPC contents among samples

*StS* stigmasteryl stearate, *StO* stigmasteryl oleate, *StL* stigmasteryl linoleate, *StLn* stigmasteryl linolenate

**Fig. 5:**
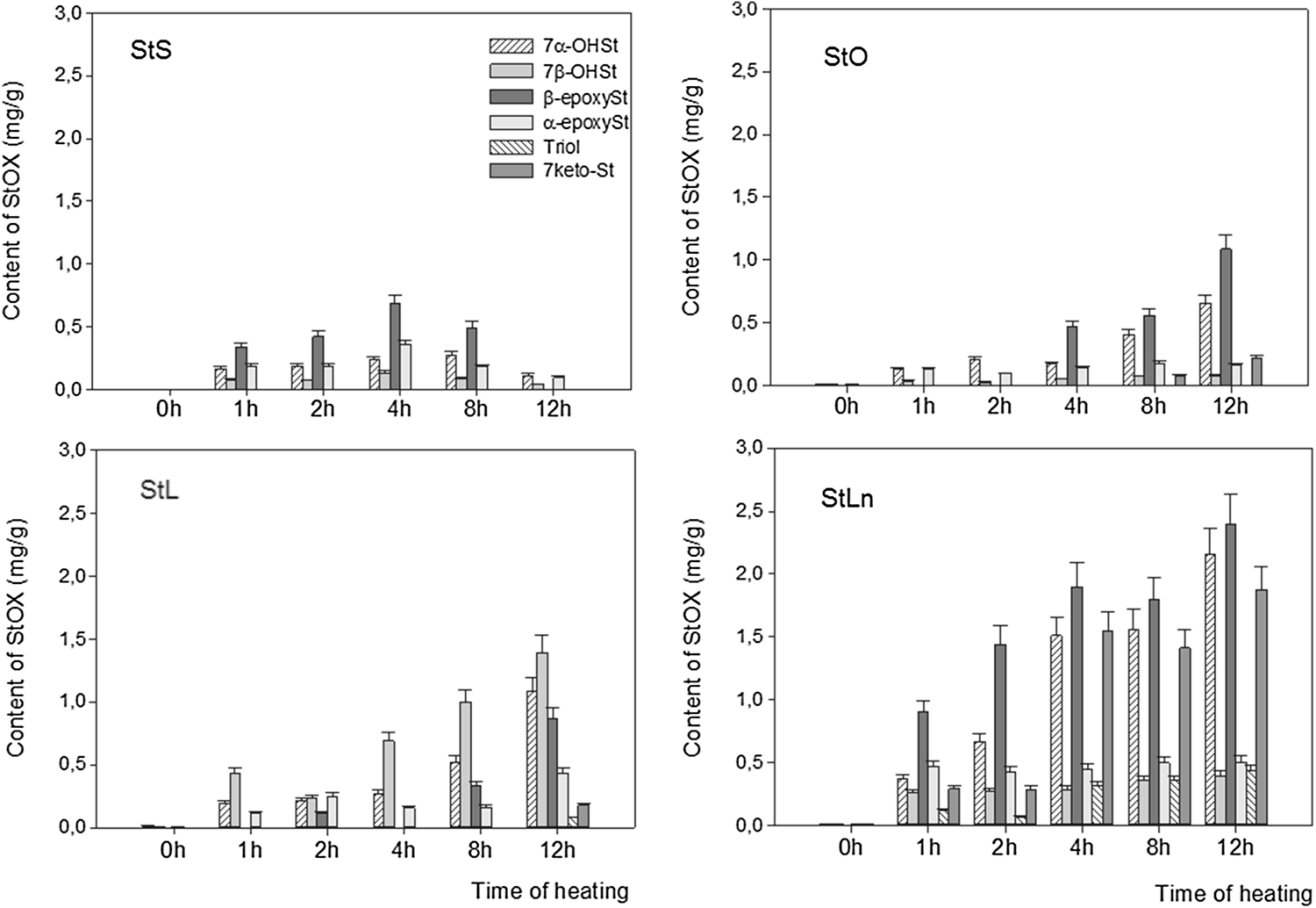
Formation of stigmasterol oxidation products during esters heated at 60 °C

**Fig. 6:**
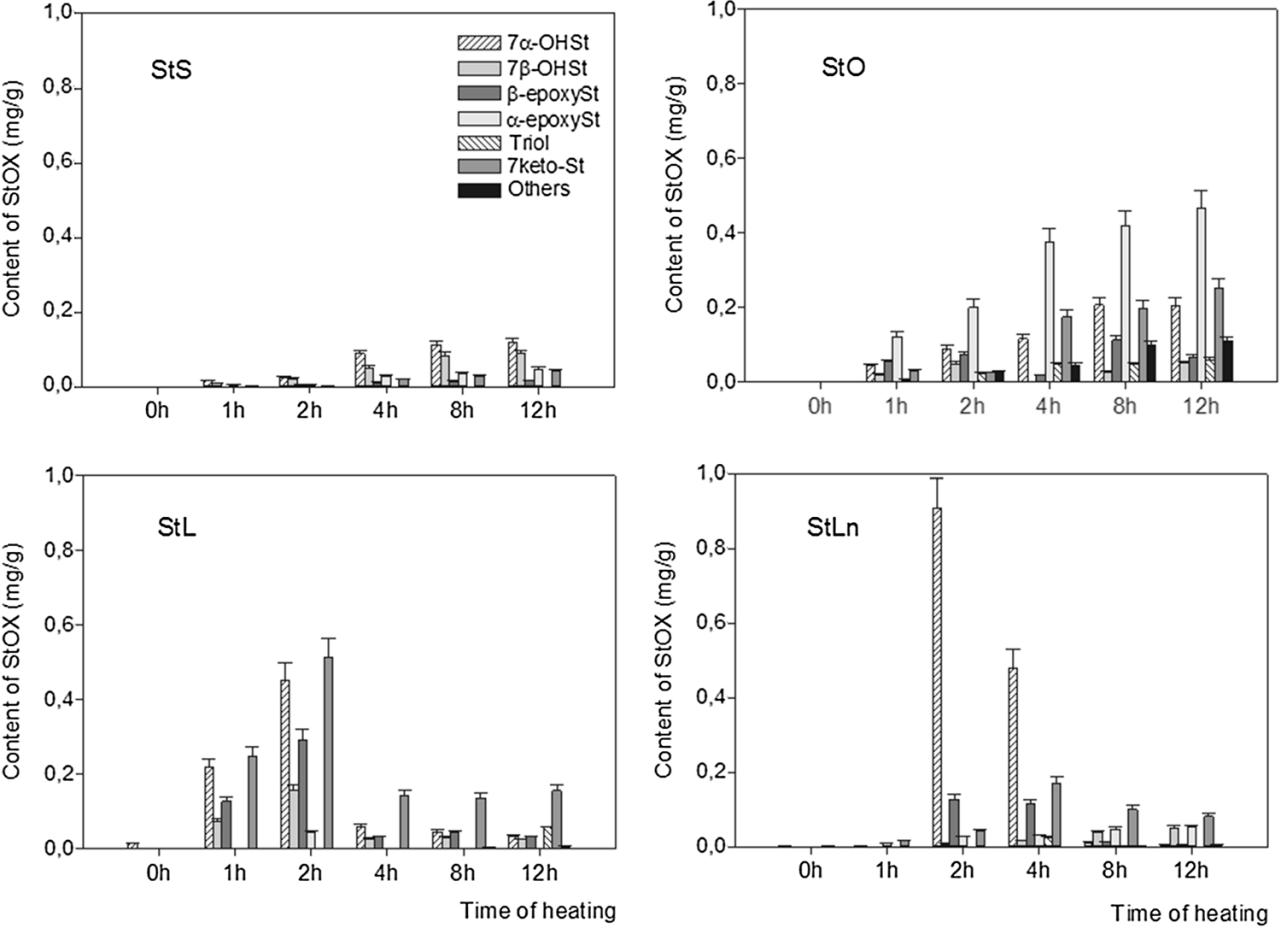
Formation of stigmasterol oxidation products during esters heated at 180 °C

During the heating of stigmasteryl esters, six oxyphytosterols were identified: 7α-hydroxystigmasterol (7αOHSt), 7β-hydroxystigmasterol (7β-OHSt), β-epoxystigmasterol (β-epoxySt), α-epoxystigmasterol (α-epoxySt), stigmastentriol (triol), and 7-ketostigmasterol (7-ketoSt). In all the unheated steryl esters, the concentration of total stigmasterol oxidation products (StOX) ranged from 0 to 0.02 mg/g. During thermal treatment of StS at 60 °C, the total content of StOX increased over 4 h of heating, followed by a slight decrease near the end of heating (Fig. 5). The same ester heated at 180 °C formed a lower level of StOX with the total StOX content increasing ten-fold up to the end of the heating time (Fig. 6). When StO was heated at 60 °C, the total level of StOX increased from 0.3 mg/g after 1 h to 2.2 mg/g after 12 h of heating (Fig. 5). When the same ester was heated at 180 °C, the formation of oxystigmasterols was at a lower rate and ranged from 0.3 mg/g after 1 h to 1.2 mg/g after 12 h (Fig. 6).

Stigmasterol linoleate heated at 60 and 180 °C for 1 h formed the same amount of StOXs (0.7 mg/g), but after 12 h of heating the amount increased to 4.0 mg/g at 60 °C and decreased to 0.3 mg/g at 180 °C (Figs. 5, 6). The total content of StOX formed during the heating of StLn at 60 °C increased at more rapid rate and was at the highest of all the heated samples. The same ester heated at 180 °C for 1 h formed only 0.03 mg/g of oxyphytosterols after 2 h, but the amount increased to 1.1 mg/g with further heating which decreased the concentration of these compounds (Fig. 6). These data show that heating at a lower temperature (60 °C) induced the formation of oxidative derivatives of stigmasterol whereas heating at 180 °C caused faster degradation and stimulated interaction. The disappearance of StOX at higher temperatures is related to the formation of oligomers and low-molecular-weight compounds, which can negatively affect sensory properties [33]. For all investigated esters, the most rapid increase in StOX contents was observed during the first 2 h of heating. This must be taken into account when food products enriched with phytosterols and its esters are recommended for use in cooking or frying.

When esters were heated at 60 °C, the main oxidation derivatives formed were β-epoxySt, followed by the 7α-OHSt. Only in StL 7β-OHSt was the main product oxyphytosterol, followed by 7α-OHSt and β-epoxySt (Fig. 5).

The dominant oxidative derivative formed during the heating of StS, StL and StLn at 180 °C was 7α-OHSt, whereas in StO, α-epoksySt was detected at the highest level (Fig. 6). Stigmasterol oxidation products formed during the heating of stigmasteryl esters were accumulated mainly in samples treated at 60 °C, whereas their presence was lower during heating at 180 °C. This finding clearly indicates the instability and potential chemical reactivity of these degradation products, which may lead to the formation of a whole range of other products, such as low-molecular-weight compounds, that could affect sensory properties. Also, as previously reported, oligomers are formed at large levels as an effect of condensation [35].

Oxyphytosterols formed during the heating of esters at 60 °C made up 0.5–4.0% of TPC for StS, 0.5–3.1% of TPC for StO, 1.3–5.7% of TPC for StL and 3.8–8.4% of TPC for StLn. At 180 °C, the share of stigmasterol oxidation products was much lower and ranged up to 0.7% (Table 3). The highest contribution of oxyphytosterols to TPC was observed in the samples heated for the shortest time again under these conditions, the oxidative degradation of stigmasterol was strongest, and the accumulation of oxidation products occurred, following their greater stability in milder conditions.

The data presented here show that the rate of thermo-oxidative change in the stigmasteryl moiety during heating at 60 °C differs from that at 180 °C. When stigmasterol and cholesterol were heated in sunflower oil, 7α-hydroxy- and 7-keto- derivatives were the most abundant oxidation products [31]. When the same phytosterols were heated in fatty acid methyl ester medium for 3 h at 180 °C, the oxidation derivatives were formed at a much slower rate [27]. The protective effect of unsaturated fatty acids on the oxidation of sterols has been proposed by Barriuso et al. [27].

## Conclusions

Thermo-oxidative degradation of phytosteryl esters is substantially affected by the unsaturation level of fatty acids and by the typical parameters that accelerate chemical reactions, such as temperature and time. The steryl moiety degrades at a slower rate than does the fatty acid moiety during heating at 60 °C; however, at 180 °C, the opposite was observed. This indicates that stigmasterol at elevated temperatures is more prone to oxidative degradation, and with it the stimulation of further degradation of ester molecules, demonstrating the free radical mechanism of degradation. Products formed during thermo-oxidative degradation of steryl esters will directly affect the nutritional quality of any food products containing these ingredients. Additionally, degradation products may directly and negatively affect human health, because, as indicated above, they are absorbed from the human gut into the blood system and are distributed through the body, replacing functional components associated with cholesterol metabolism. The results of this experiment further validate the research required to determine the nutritional and metabolic effects of these degradation products, which are formed in food products and enriched during preparation, storage and processing.

## References

[1] Moreau RA, Whitaker BD, Hicks KB (2002). Phytosterols, phytostanols, and their conjugates in foods: structural diversity, quantitative analysis, and health promoting uses. Prog Lipid Res.

[2] Moreau RA, Dutta PC (2004). Phytosterols as functional food components and nutraceuticals.

[3] Judd JT, Baera DJ, Chen SC, Clevidence BA, Muesing RA, Kramer M, Meijer GW (2002). Plant sterol esters lower plasma lipids and most carotenoids in mildly hypercholesterolemic adults. Lipids.

[4] Downs JR, Clearfield M, Weis S, Shapiro DR, Beere PA, Langendorfer A, Stein EA, Kruyer W, Gotto AM, Phil D (1998). Primary prevention of acute coronary events with lovastatin in men and women with average cholesterol levels: results of AFCAPS/TexCAPS. JAMA.

[5] Hallikainen MA, Uusitupa MIJ (1999). Effect of 2 low-fat stanol ester containing margarines on serum cholesterol concentrations as part of a low-fat diet in hypercholesterolemic subjects. Am J Clin Nutr.

[6] Vanhanen HT, Blomqvist S, Ehnholm C, Hyvonen M, Jauhiainen M, Torstila I, Miettinen TA (1993). Sitostanol ester in dietary oil reduces serum cholesterol. Effect on serum plant sterols and cholesterol precursors. J Lipid Res.

[7] Lichtenstein AH, Deckelbaum RJ (2001). Stanol/sterol ester-containing foods and blood cholesterol levels. AHA Science Advisory. A statement for healthcare professionals from the nutrition committee council on nutrition, physical activity, and metabolism of the american heart association. Circulation.

[8] Berger A, Jones PJH, Abumweis SS (2004). Plant sterols: factors affecting their efficacy and safety as functional food ingredients. Lipids Health Dis.

[9] Garcia-Llatas G, Rodriguez-Estrada MT (2011). Current and new insights on phytosterols oxides in plant sterol-enriched food. Chem Phys Lipids.

[10] Rudzińska M, Przybylski R, Wąsowicz E (2009). Products formed during thermo-oxidative degradation of phytosterols. J Am Oil Chem Soc.

[11] Gunawan S, Fabian C, Ju YH (2008). Isolation and purification of fatty acid steryl esters from soybean oil deodorizer distillate. Ind Eng Chem Res.

[12] Hirota Y, Nagao T, Watanabe Y, Nakai S, Kitano M, Sugihara A, Shimada Y (2003). Purification of steryl esters from soybean oil deodorizer distillate. J Am Oil Chem Soc.

[13] Neil HAW, Meijer GW, Roe LS (2001). Randomised controlled trial of use by hypercholesterolaemic patients of a vegetable oil sterol-enriched fat spread. Atherosclerosis.

[14] Houhoula DP, Oreopoulou V, Tzia C (2003). The effect of process time and temperature on the accumulation of polar compounds in cottonseed oil during deep-fat frying. J Sci Food Agric.

[15] Firestone D, Stier RF, Blumenthal MM (1991). Regulation of frying fats and oils. Food Technol.

[16] Emport LLC (2017) White paper: monitoring polar compounds in fryer oil. https://emportllc.com/wp-content/uploads/2013/02/White-Paper-Monitoring-polar-compounds-in-fryer-oil.pdf. Accessed 03 Mar 2017

[17] Tomoyori H, Kawata Y, Higuchi T, Ichi I, Sato H, Sato M, Ikeda I, Imaizumi K (2004). Phytosterol oxidation products are absorbed in the intestinal lymphatics in rats but do not accelerate atherosclerosis in apolipoprotein E-deficient mice. J Nutr.

[18] O’Callaghan Y, McCarthy FO, O’Brien NM (2014). Recent advances in phytosterols oxidation products. Biochem Biophys Res Commun.

[19] Weingärtner O, Baber R, Teupser D (2014). Plant sterols in food: no consensus in guidelines. Biochem Biophys Res Commun.

[20] Neises B, Steglich W (1978). Simple method for esterification of carboxylic acid. Angew Chem Int Edit.

[21] Barnsteiner A, Lubinus T, di Gianvito A, Schmid W, Engel K-H (2011). GC-based analysis of plant stanyl fatty acid esters in enriched foods. J Agric Food Chem.

[22] Firestone D (ed) (1997) Determination of the composition of the sterol fraction of animal and vegetable oils and fats by TLC and capillary GLC. AOCS Official Method Ch 6-91. AOCS, Champaign, IL

[23] AOCS Official Method Ce 1 k-07 (2007). Direct methylation of lipids for the determination of total fat, saturated, *cis*-monounsaturated, *cis*-polyunsaturated, and *trans* fatty acids by chromatography.

[24] DGF Standard Method CIII 3e (06): Polar compounds in frying oils. Rapid method with micro silica gel column. In: Deutsche Einheitsmethoden zur Untersuchung von Fetten. Fettprodukten. Tensiden und verwandten Stoffen, Wissenschaftliche Verlagsgesellschaft, Stuttgart, Germany 2013

[25] Rudzińska M, Przybylski R, Wąsowicz E (2014). Degradation of phytosterols during storage of enriched margarines. Food Chem.

[26] Thurnhofer S, Vetter W (2005). A gas chromatography/electrol ionization-mass spectrometry-selected ion monitoring method for determining the fatty acid pattern in food after formation of fatty acid methyl esters. Food Chem.

[27] Barriuso B, Astiasarán I, Ansorena D (2016). Unsaturated lipid matrices protect plant sterols from degradation during heating treatment. Food Chem.

[28] Ansorena D, Barriuso B, Cardenia V, Astiasarán I, Lercker G, Rodrigues-Estrada M (2013). Thermo-oxidation of cholesterol. Effect of the unsaturation degree of the lipid matrix. Food Chem.

[29] Soupas L, Huikko L, Lampi A-M, Piironen V (2005). Esterification affects phytosterol oxidation. Eur J Lipid Sci Technol.

[30] Lehtonen M, Lampi A-M, Riuttamäki M-A, Piironen V (2012). Oxidation reactions of steryl esters in a saturated lipid matrix. Food Chem.

[31] Barriuso B, Ansorena D, Poyato C, Astiasaran I (2015). Cholesterol and stigmasterol within a sunflower oil matrix: thermal degradation and oxysterols formation. Steroids.

[32] Márquez-Ruiz G, Dobarganes MC, Perkins EG, Erickson MD (1996). Deep frying chemistry, nutrition and practical applications.

[33] Rudzińska M, Przybylski R, Zhao YY, Curtis JM (2010). Sitosterol thermo-oxidative degradation leads to the formation of dimers, trimers and oligomers—a study using combined size exclusion chromatography/mass spectrometry. Lipids.

[34] Velasco J, Marmesat S, Marquez-Ruiz G, Dobarganes MC (2004). Formation of short-chain glycerol-bound oxidation products and oxidised monomeric triacylglycerols during deep-frying and occurrence in used frying. Eur J Lipid Sci Technol.

[35] Sosińska E, Przybylski R, Aladedunye F, Hazendonk P (2014). Spectroscopic characterisation of dimeric oxidation products of phytosterols. Food Chem.

